# AtomNet-Aided OTUD7B Inhibitor Discovery and Validation

**DOI:** 10.3390/cancers15020517

**Published:** 2023-01-14

**Authors:** Jianfeng Chen, Derek L. Bolhuis, Christian Laggner, Deyu Kong, Le Yu, Xiaodong Wang, Michael J. Emanuele, Nicholas G. Brown, Pengda Liu

**Affiliations:** 1 Lineberger Comprehensive Cancer Center, The University of North Carolina at Chapel Hill, Chapel Hill, NC 27599, USA; 2 Department of Biochemistry and Biophysics, The University of North Carolina at Chapel Hill, Chapel Hill, NC 27599, USA; 3 Atomwise Inc., San Francisco, CA 94103, USA; 4 Center for Integrative Chemical Biology and Drug Discovery, Division of Chemical Biology and Medicinal Chemistry, Eshelman School of Pharmacy, The University of North Carolina at Chapel Hill, Chapel Hill, NC 27599, USA; 5 Department of Pharmacology, The University of North Carolina at Chapel Hill, Chapel Hill, NC 27599, USA

**Keywords:** OTUD7B, small molecule inhibitor, NSCLC, leukemia, cell proliferation

## Abstract

**Simple Summary:**

Protein deubiquitinases are an important family of enzymes commonly deregulated in cancer. However, few deubiquitinase inhibitors have been identified and used in cancer treatment. Here, using an artificial intelligence (AI) AtomNet guided screening pipeline, we searched 4 million compounds for potential OTUD7B inhibitors and validated one compound with a series of in vivo and in vitro assays. This compound, 7Bi, efficiently inhibited OTUD7B activity both in cells and in vitro, leading to reduced cancer cell proliferation. These data show a promise to use 7Bi in treating cancers with increased OTUD7B expression including both breast and lung cancer.

**Abstract:**

Protein deubiquitinases play critical pathophysiological roles in cancer. Among all deubiquitinases, an oncogenic function for OTUD7B has been established in genetic NSCLC murine models. However, few deubiquitinase inhibitors have been developed due to technical challenges. Here, we report a putative small molecule OTUD7B inhibitor obtained from an AI-aided screen of a 4 million compound library. We validated the effects of the OTUD7B inhibitor (7Bi) in reducing Akt-pS473 signals in multiple NSCLC and HEK293 cells by blocking OTUD7B-governed GβL deubiquitination in cells, as well as inhibiting OTUD7B-mediated cleavage of K11-linked di-ub in an in vitro enzyme assay. Furthermore, we report in leukemia cells, either genetic depletion or 7Bi-mediated pharmacological inhibition of OTUD7B reduces Akt-pS473 via inhibiting the OTUD7B/GβL signaling axis. Together, our study identifies the first putative OTUD7B inhibitor showing activities both in cells and in vitro, with promising applications as a therapeutic agent in treating cancer with OTUD7B overexpression.

## 1. Introduction

Protein ubiquitination plays critical roles in regulating protein function including protein stability, trafficking, binding partners, cellular localization, and others [1]. Protein ubiquitination is a result of a balanced action from both ubiquitination and deubiquitination, governed by E3 ubiquitin ligases and deubiquitinases (DUBs), respectively. There are ~100 deubiquitinases in mammals [2] with either cysteine protease or metalloprotease activities, largely divided into seven families including USPs (ubiquitin-specific proteases), OTUs (ovarian tumor proteases), UCHs (ubiquitin C-terminal hydrolases), Josephins (machado-josephin domain proteases), MINDYs (MIU containing novel DUB family), JAMMs (Jab1/Mov34/Mpr1 Pad1 N-terminal proteases), and lastly ZUFSP (zinc finger with UFM1-specific peptidase) [3,4,5]. Among these DUB members, the OTU family of DUBs has been tightly connected with both ubiquitin linkage specificity and cancer relevance. For example, multiple studies support the oncogenic role of OTUB1 in tumorigenesis. Specifically, OTUB1 prevents ER-associated degradation of PD-L1 to contribute to immune suppression [6], inhibits Ras mono-ubiquitination to trigger lung cancer development [7], promotes RohA activation to induce prostate cancer metastasis [8], stabilizes ATF6 to facilitate bladder cancer growth [9], deubiquitinates and stabilizes FOXM1 to promote renal cell carcinoma progression [10], interacts with and stabilizes SLC7A11 to regulate ferroptosis [11], and stabilizes c-Myc to facilitate Myc-dependent multiple myeloma proliferation [12]. On the other hand, the roles of OTUD3 are cancer-type dependent. Specifically, OTUD3 deubiquitinates and stabilizes PTEN [13] and p53 [14] to suppress breast tumorigenesis, but it stabilizes GRP78 to promote lung tumorigenesis [15]. In addition, OTUD3 differentially regulates nucleic acid sensing pathways; OTUD3 removes K63-linked ubiquitin moieties from MAVS to suppress MAVS activation in response to cytosolic RNA challenges [16] while deubiquitinating K48-ubiqutinated cGAS to stabilize cGAS thus potentiating DNA sensing [17]. OTUD3 also deubiquitinates and stabilizes PPARδ to regulate energy metabolism and obesity [18]. 

In addition, an oncogenic function of OTUD7B in cancer has been well demonstrated. OTUD7B removes K63-linked ubiquitination on GβL to promote mTORC2 activation in facilitating NSCLC progression [19]. This effect is largely mediated by the major mTORC2 downstream target Akt. Akt is an oncogenic kinase hyperactivated in virtually all types of cancer [20]. The mTORC2/GβL kinase complex phosphorylates Akt-Ser473 to promote the full activation of Akt together with PDK1-mediated Akt-Thr308 phosphorylation. Thus, Akt-pS473 is a faithful marker for mTORC2 activation and OTUD7B-mediated GβL deubiquitination in cells. Notably, OTUD7B-induced cleavage of K63-linked chains on GβL does not affect GβL’s protein stability, but rather shifts GβL from mTORC1 to mTORC2, leading to subsequent mTORC2 activation [19], antagonizes APC/C-mediated K11-linked substrate ubiquitination and proteolysis [21], deubiquitinates LSD1 to regulate its binding with co-REST and genome-wide occupancy to fuel breast cancer metastasis [22], stabilizes ERα to promote breast cancer progression [23], and also regulates HIF-1a homeostasis in a proteasome-independent manner in renal cancer [24]. Notably, multiple ubiquitin linkages including K63 and K11, have been shown to be regulated by OTUD7B. In addition, OTUD7B participates in immune regulation. OTUD7B deubiquitinates and stabilizes TRAF3 to induce non-canonical NF-κB activation in immune regulation [25], deubiquitinates and stabilizes Zap70 in T cell activation [26], and removes K63-linked ubiquitin chains on p62 to enhance its oligomerization that promotes IRF3 degradation as a negative feedback mechanism to restrain IFN production [27]. Increased OTUD7B expression is observed in breast cancer [28] and lung cancer [29]. OTUD7B is a prognostic maker in diffuse large B-cell lymphoma [30] and NSCLC [31], as well as a potential therapeutic target for myocardial infarction by ameliorating fibrosis [32], and predicts poor responses to paclitaxel in TNBC [28]. Considering its oncogenic function in cancer and its great potential as a cancer drug target but without available inhibitors, here we aim to rely on artificial intelligence AtomNet to screen for small molecule OTUD7B inhibitors.

## 2. Materials and Methods

### 2.1. Cell Lines and Cell Culture

A549, H358, H520, H1299, HEK293T and HEK293 were cultured in DMEM medium supplemented with 10% FBS, 100 U penicillin and 100 mg/mL streptomycin. Acute myeloid leukemia cell lines K562, HL60 and leukemia monocytic cell line THP1 were cultured in RPMI 1640 medium supplemented with 10% FBS, 100 U penicillin and 100 mg/mL streptomycin. All the cell lines were cultured in a 37 °C incubator with 5% CO_2_. HEK293 and HEK293T cells were purchased from the UNC Lineberger Tissue Culture Facility. A549, H358, H520 and H1299 lung cancer cells were obtained from Dr. Chad Pecot’s lab at UNC. THP1 cells were obtained from Dr. Albert Baldwin’s lab at UNC. K562 and HL60 cells were obtained from Dr. Greg Wang’s lab at UNC.

### 2.2. Antibodies

All antibodies were used at a 1:2000 dilution in TBST buffer with 5% non-fat milk and incubated at 4 °C with gentle shaking overnight for Western blotting. Anti-HA antibody (3724), anti-p-AKT (Ser473) (4060), anti- anti-p-p70 S6 Kinase (Thr389) (9234), anti-p-NDRG1 (Thr346) (89166), anti-p-GSK-3β (Ser9) (5558), anti-p-FoxO1 (Thr24)/FoxO3a (Thr32) antibody (9464), anti-K63-linkage specific polyubiquitin antibody (12930), anti-GβL antibody (3274), anti-Rictor antibody (2114), anti-Sin1 antibody (12860), anti-mTOR antibody (2983), anti-AKT (4691), anti-rabbit IgG, HRP-linked antibody (7074) and anti-mouse IgG, HRP-linked antibody (7076) were obtained from Cell Signaling Technology. Anti-vinculin antibody (sc-25336) was obtained from Santa Cruz Biotechnology. Monoclonal anti-Flag antibody (F-3165, clone M2) and anti-α-tubulin antibody (T-5168) were obtained from Sigma. Anti-OTUD7B antibody (16605-1-AP) was obtained from Proteintech. 

### 2.3. Plasmids

HA-GβL, His-Ub and Flag-OTUD7B plasmids were constructed as described [19]. shRNA sequences used for OTUD7B depletion were as follows.

shOTUD7B-23: GCAAGGAGGCTAAACAAAGTT
shOTUD7B-77: CAAAGTTAAGCTCAACTAATT
shOTUD7B-95: TGGAAATGCTCACGGTTTATA

### 2.4. Immunoblot and Immunoprecipitations Analyses

Cells were harvested and lysed in EBC buffer (50 mM Tris pH 7.5, 120 mM NaCl, 0.5% NP-40) supplemented with protease inhibitor cocktail (EDTA-free, mini-tablet) (Bimake) and phosphatase inhibitor cocktail (Bimake) by rotating in a cold room for 10 min followed by high-speed centrifugation at 4 °C. The protein concentrations in the supernatants of whole cell lysates were measured by NanoDrop OneC using the Bio-Rad Bradford protein assay reagent. Equal amounts of whole cell lysates were resolved on SDS-PAGE and immunoblotted with indicated antibodies. For immunoprecipitation analysis, 1 mg of total cell lysate was incubated with the indicated beads for 3–4 h at 4 °C. The recovered immuno-complexes were washed thoroughly three times with NETN buffer (20 mM Tris, pH 8.0, 100 mM NaCl, 1 mM EDTA and 0.5% NP-40) before being resuspended in SDS sample buffer and resolved on SDS-PAGE and immunoblotted with indicated antibodies. 

### 2.5. Colony Formation Assays

Five-hundred cells from OTUD7B depleted A549 cells were seeded into each well of 6-well plates and cultured in the 37 °C incubator with 5% CO2 for 10–15 days until the formation of visible colonies. Triplicates were performed. To test the effect of a given inhibitor on colony formation ability, 800 cells from A549, H520 and H1299 cell lines were seeded into each well of 6-well plates, respectively. Each inhibitor was added at the indicated concentration the next day. Colonies were counted after 10 days of treatment. The colony staining method was as described [33]. Briefly, the medium was removed, and colonies were fixed with fixation solution (20% acidic acid, 10% methanol) for at least 30 min and stained with 1.0% crystal violet overnight. Colonies were then washed several times with distilled water and air-dried. Colony numbers were counted manually. Three independent experiments were performed.

### 2.6. Cell Viability and Proliferation Assay

Two thousand cells from indicated NSLC cell lines were seeded into each well of 96-well plates. Cell viability was measured 3 days post-treatment using MTT assays following the manufacturer’s instruction. For cell viability assays with inhibitor treatment, 2000 cells from indicated NSLC and HEK293 cell lines were seeded in 96-well plates. An inhibitor was added the next day at indicated concentration and cell viability was measured after 3-day treatment. For cell proliferation assays in THP1, K562 and HL60 cell lines, 1 × 10^6^ cells from each indicated cell line were seeded in a 12.5 cm^2^ cell culture flask and cells were counted by a hemocytometer under a microscope at the indicated time to monitor cell proliferation. To test inhibitor effects on THP1, K562 and HL60 cell growth, 2 × 10^5^ cells were seeded in a 12.5 cm^2^ cell culture flask. The inhibitor was added the next day at indicated concentrations and the cell number was counted after 3 days of treatment. Three independent experiments were performed to generate error bars.

### 2.7. Ubiquitin Chain Cleavage Assay

OTUD7B (residues 56–446), OTUD7A (residues 1–462) [34], USP21 (residues 196–565), and USP9X (residues 1551–1970) proteins were expressed in *E. coli* BL21-Codon Plus (DE3)-RIL cells. Through an N-terminal GST tag, the DUBs were purified by affinity chromatography using glutathione sepharose 4B (GS4B) resin. Following the liberation of the DUB from the GST tag, the DUBs were subjected to size exclusion chromatography (SEC). To form the K11-linked di-ub, individual ubiquitins (K11R-substituted donor Ub and ΔGG acceptor ubiquitin) were purified and fluorescently labeled on an N-terminal cysteine using either Cy3- (donor) or Cy5-maleimide (acceptor). The different ubiquitins were then added to a mixture containing UBA1, UBE2S, and MgATP to form di-ub. After overnight incubation at room temperature, UBE2S was removed by cation-exchange chromatography and the di-ub was purified by SEC. These proteins were used to monitor DUB activity in the presence or absence of various inhibitors. The inhibitors were incubated with DUBs in a reaction buffer (20 mM Hepes pH = 8.0, 200 mM NaCl, 0.25 mg/mL BSA and 0.005% Tween-20) for 5 min at room temperature and then 0.5 μM fluorescently labeled di-ub (K11-linked) was added to start the reaction. 5X SDS-PAGE loading buffer was used to quench the reaction at the indicated time point. Samples were run on SDS-PAGE and fluorescently scanned using an Amersham^TM^ Typhoon^TM^ Biomolecular Imager. Mono-ubiquitin was quantified to generate an IC50 value. Three independent experiments were performed to generate the error bars.

### 2.8. Ubiquitin-AMC Assay

Fifty micromoles of 7Bi were incubated with 300 nM OTUD7B (residues 56–446), 10 μM OTUD7A (residues 1–462), 200 nM USP21 (residues 196–565), or 500 nM USP9X (residues 1551–1970) in reaction buffer (20 mM HEPES pH = 8.0, 200 mM NaCl, 0.25 mg/mL BSA and 0.005% Tween-20) for 5 min at room temperature. Then, 2 μM ubiquitin-AMC was added to start the reaction. The fluorescence emission data were collected at an excitation wavelength of 355 nm and emission wavelength of 460 nm every 3 min. Reactions were completed three times to generate the error bars.

### 2.9. Compound Purification

7Bi was purified by recrystallization from methanol and the purity was higher than 99%. The purity of this compound was determined by LC−MS. LC/MS and was performed using an analytical instrument with a UV detector set to 220 nm, 254 nm, and 280 nm, and a single quadrupole mass spectrometer using an electrospray ionization (ESI) source. The sample was injected (10 μL) onto a 4.6 × 50 mm, 1.8 μM, C18 column at room temperature. A linear gradient from 10% to 100% B (Acetonitrile + 0.1% acetic Acid) in 6.0 min was followed by pumping 100% B for another 2 or 4 min with A being H2O + 0.1% acetic acid. The flow rate was 1.0 mL/min.

### 2.10. AtomNet-Based Small Molecule Virtual Screen

The procedure is as described previously [35]. The virtual screen for new small molecule inhibitors of OTUD7B was performed using Atomwise’s proprietary AI-based AtomNet^®^ screening platform. We employed a single global AtomNet model to predict the binding affinity of small molecules to a target protein. The catalytic OTU domain of OTUD7B has been crystallized in complex with a diubiquitin, monoubiquitin, and as an apo structure PDB codes 5LRV, 5LRW, 5LRU, respectively [36]. As in our previous paper on OTUD7A, the area of the distal S1 ubiquitin-binding site was chosen due to the structural differences between OTUD7B and its close homolog OTUD7A to achieve potential ligand selectivity. The monoubiquitin-bound OTUD7B crystal structure 5LRW (after removal of the monoubiquitin), which had also served as the homology template for our previous work on OTUD7A, was chosen for the virtual screen, with the binding site located between residues W234, Q237, Q238, T239, Q241, N242, S245, G246, L247, Y249, W254, E257, W258, E260, L261, L264, L294, E295, E296, F297, H298, P330, F331, H390, and F391. 

We again screened a filtered version of the Mcule small-molecule library (v20171018, 4,025,533 compounds). Compounds were docked and scored with the AtomNet model and ranked by their scores. After postprocessing, a set of 59 compounds containing diverse chemical scaffolds was selected from the top-scoring compounds and sourced from Mcule. Each compound was dissolved in DMSO with a concentration of 10 mM. All 10 μM DMSO stocks were analyzed by Mcule for purity via LC-MS. Only compounds that passed ≥85% purity were shipped and tested. The compound samples were assayed in a blinded way (chemical identities unknown to the lab researcher, with two negative control samples containing pure DMSO mixed in). 

### 2.11. Statistical Analysis

Statistical analysis was performed as described previously [35]. Briefly, SPSS 11.5 Statistical Software was used for statistical analyses. Asterisks (*p* ≤ 0.05) labeled statistical significance. The results are shown as means ± SD from independent experiments as indicated in figure legends. One-way ANOVA was used to evaluate differences between control and experimental conditions.

## 3. Results

### 3.1. Artificially Intellegence (AI)-Aided Virtual Screening for OTUD7B Small Moleule Inhibitors

**Figure 1:**
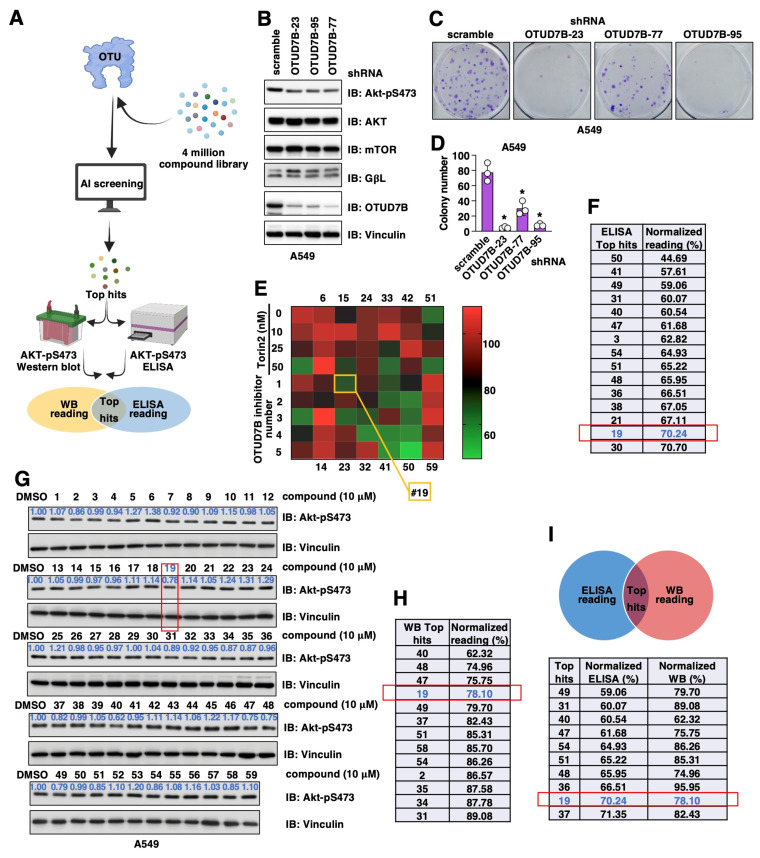
Searching for OTUD7B small molecule inhibitors. (**A**) A cartoon illustration for the pipeline of searching for OTUD7B small molecule inhibitors. AtomNet was used to search a 4 million small molecule library for hits fitting into the OTUD7B-OTU structure. Top hits were purchased and effects of OTUD7B inhibition by these small molecules were tested by both Akt-pS473 ELISA and Western blotting in A549 cells. Overlapping hits from two assays were identified and tested further. (**B**) Immunoblot (IB) analyses of whole cell lysates (WCL) derived from control or OTUD7B depleted A549 cells. (**C**) Representative colony formation analyses images using A549 cells obtained in (**B**) and quantified in (**D**). Error bars were calculated as mean+/-SD, *n* = 3. * *p* < 0.05 (one-way ANOVA test). (**E**) A heatmap illustration of Akt-pS473 ELISA results obtained from treating A549 cells with 10 μM of each indicated chemical for 16 h. (**F**) A list of chemicals with the ability to suppress 30% Akt-pS473 signals from assays in (**E**). (**G**) IB analyses of WCL from A549 cells treated with indicated compounds for 16 h. (**H**) A list of chemicals with the ability to suppress 10% Akt-pS473 signals from assays in (**G**). (**I**) A list of overlapped hits from both (**F**,**H**).

Previously, we reported OTUD7A as a vulnerability in Ewing sarcoma by deubiquitinating and stabilizing EWS-FLI1; we have successfully utilized the AtomNet model [37] to identify 7Ai as an OTUD7A catalytic inhibitor [35]. Relying on a solved crystal structure of the OTUD7B catalytic OTU domain (PDB: 5LRW), we tested 4 million commercially available, drug-like compounds fitting into this structure in a virtual screen and purchased and tested a diverse set of 59 compounds from the top-scoring virtual hits (Figure 1A).

### 3.2. Validation and Selection of Viable OTUD7B Inhibitors in Cells

Considering previous genetic murine models and cell line-based studies firmly demonstrated that genetic OTUD7B depletion attenuated NSCLC (non-small cell lung cancer) tumor progression largely through inhibiting the OTUD7B/GβL/Akt-pS473 signaling [19], we used NSCLC cells to examine effects of these 59 synthesized small molecules in suppressing Akt-pS473 signals. We first validated that in the NSCLC A549 cells, the depletion of endogenous OTUD7B reduced Akt-pS473 (Figure 1B), leading to subsequently reduced A549 cell growth in vitro (Figure 1C,D). Next, we used two approaches to search for possible OTUD7B inhibitors by monitoring Akt-pS473 changes including Akt-pS473 ELISA and Western blotting in A549 cells treated with each of these 59 small molecules for 16 h at a concentration of 10 μM (Figure 1A). In Akt-pS473 ELISA assays using Torin 2 (a pan-mTOR inhibitor) as a positive control and DMSO as a negative control (Figure 1E), we found 15 compounds with an inhibitory ability above 30% (Figure 1F). From the Akt-pS473 Western blotting approach, we found 13 compounds suppressed phosphorylation by more than 10% (Figure 1G,H). Overlaying hits from both of these in-cell validation approaches led to the identification of 10 common hits (Figure 1I).

### 3.3. Validation of OTUD7B Inhibitors in Multiple NSCLC Cell Lines

**Figure 2:**
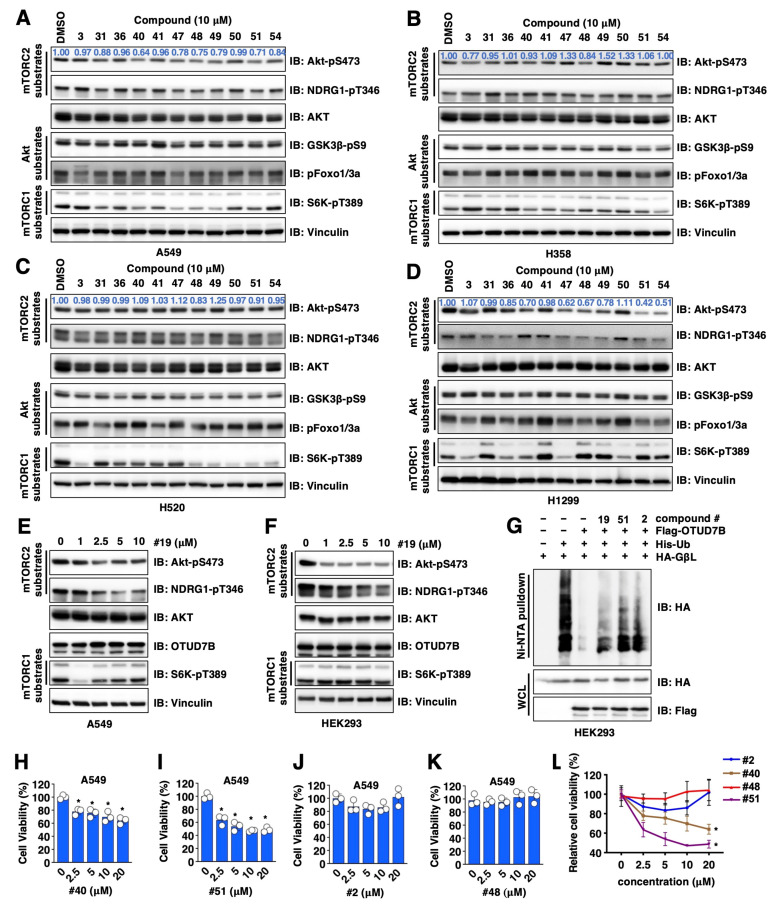
Validation for OTUD7B small molecule inhibitors in cells. (**A**–**D**) IB analyses of WCL from indicated NSCLC cells treated with 10 μM of indicated compounds for 16 h. (**E**,**F**) IB analyses of WCL from either A549 (**E**) or HEK293 (**F**) cells treated with indicated doses of compound #19 for 16 h. (**G**) IB analyses of Ni-NTA pulldowns and WCL from HEK293 cells transfected with indicated DNA constructs. Where indicated, cells were treated with 10 μM of indicated compounds for 16 h before cell collection. (**H**–**L**) Quantifications of cell viability by MTT assays in A549 cells treated with indicated doses of indicated compounds for 72 h. Error bars were calculated as mean ± SD, *n* = 3. * *p* < 0.05 (one-way ANOVA test).

Considering the poised genetic composition from a single cell line may cause the biased selection of OTUD7B inhibitors, as well as the regulation of Akt-pS473 through OTUD7B-independent mechanisms, we further tested the effects of 10 common hits identified from both Akt-pS473 ELISA and Western blotting (Figure 1I) in additional NSCLC cell lines, including A549 (Figure 2A and Figure S1A), H358 (Figure 2B), H520 (Figure 2C and Figure S1B) and H1299 (Figure 2D and Figure S1C) at a single dose of 10 μM for 16 h. Among them, compounds #2 (Figure S1A–E), #40 (Figure 2A–D and Figure S1F), #51 (Figure 2A–D) and #19 (Figure 2E and Figure S1G–I) showed effects in suppressing the Akt-pS473 signal in at least three out of four NSCLC lines tested in both compound dose- (Figure S1D,F–I) and time- (Figure S1E) dependent manners. In addition, compound #19 also efficiently suppressed Akt-pS473 in HEK293 cells (Figure 2F), a cell line commonly used in studying the regulation of mTOR signaling [38].

To further reveal if OTUD7B inhibitor-mediated suppression of Akt-pS473 is largely due to changes associated with OTUD7B-governed GβL ubiquitination, we examined the effects of these compounds in modulating GβL ubiquitination in in-cell ubiquitination assays and found that compounds #19, #52 and #2 partially blocked OTUD7B-induced GβL deubiquitination (Figure 2G). Although these compounds reduced Akt-pS473 (which earmarks Akt activation to promote cell proliferation [20]), we found unlike compounds #40 and #51 that suppressed A549 cell proliferation (Figure 2H,I), compounds #2 and #48 failed to do so (Figure 2J–L). These data suggest that in addition to regulating OTUD7B/GβL/Akt-pS473 signaling, compounds #2 and #48 may also exert OTUD7B-independent effects in regulating cell proliferation, thus they were removed from our hit list but only serve as additional negative controls.

### 3.4. Validation of OTUD7B Inhibitors through In Vitro Deubiquitination Assays

**Figure 3:**
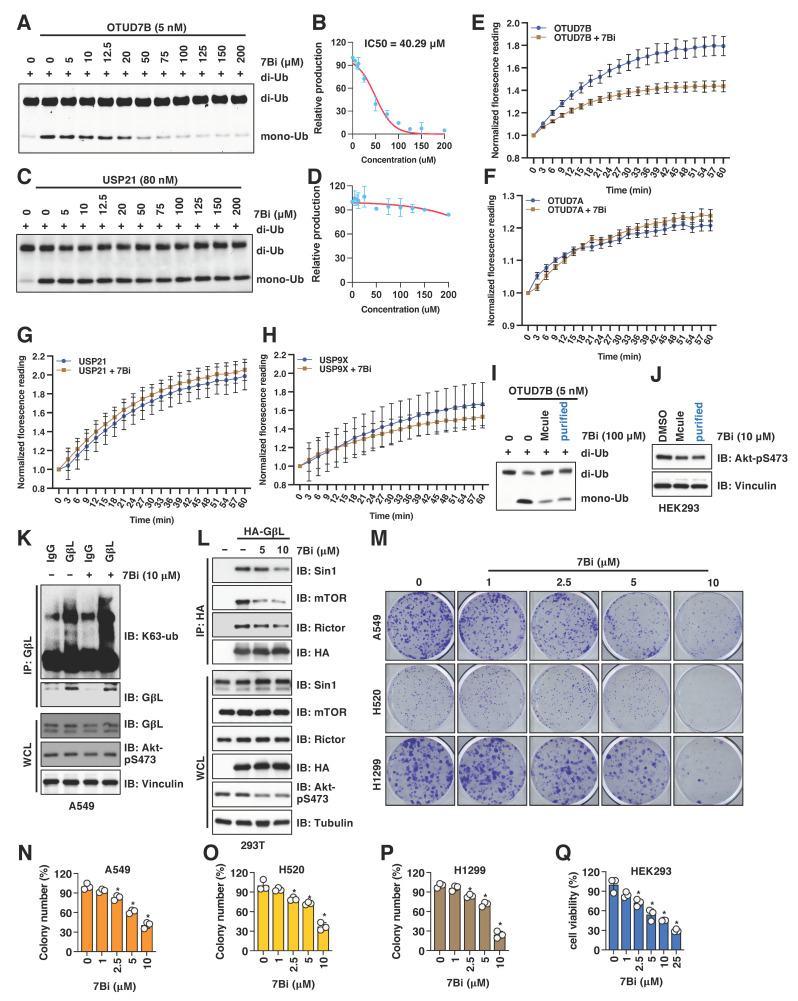
7Bi inhibits OTUD7B activity in vitro and suppress cell growth in NSCLC cells. (**A**,**B**) A representative gel image (**A**) for the in vitro OTUD7B deubiquitination assay incubating 5 nM of recombinant OTUD7B with 0.5 μM of K11-linked di-ub for 10 min in the presence of indicated doses of 7Bi. Mono-ub was quantified for IC50 value determination (**B**). (**C**,**D**) A representative gel image (**C**) for the in vitro USP21 deubiquitination assays incubating 80 nM of recombinant active USP21 proteins with 0.5 μM of K11-linked di-ub for 10 min in the presence of indicated doses of 7Bi. Mono-ub was quantified for IC50 value determination (**D**). (**E**–**H**) In vitro deubiquitination assays using ubiquitin-AMC as a substrate and indicated deubiquitinases. Normalized reading vs. reaction time periods was plotted. (**I**) A representative gel image for the in vitro OTUD7B deubiquitination assay incubating 5 nM of recombinant active OTUD7B proteins with 0.5 μM of K11-linked di-ub for 10 min in the presence of 100 μM of 7Bi (from either Mcule (90% purity) or recrystallized (99% purity)). (**J**) IB analysis of WCL derived from HEK293 cells treated with 10 μM of 7Bi (from either Mcule (90% purity) or recrystallization-purified (99% purity)). (**K**) IB analyses of GβL-IP and WCL from A549 cells treated with control or 10 μM 7Bi for 16 h. (**L**) IB analyses of HA-IP and WCL from HEK293 cells transfected with indicated DNA constructs. Where indicated, indicated doses of 7Bi were used to treat cells for 16 h before cell collection. (**M**) Representative colony formation images from indicated NSCLC cells treated with indicated doses of 7Bi once for 10 days and quantified in (**N**–**P**). Error bars were calculated as mean ± SD, *n* = 3. * *p* < 0.05 (one-way ANOVA test). (**Q**) Quantifications of cell viability by MTT assays in HEK293 cells treated with indicated doses of indicated compounds for 72 h. Error bars were calculated as mean ± SD, *n* = 3. * *p* < 0.05 (one-way ANOVA test).

To provide direct evidence for the inhibitory effects of these compounds on OTUD7B enzyme activity, we performed OTUD7B in vitro deubiquitinase assays using K11-linked di-ub as a substrate as previously reported [21]. In this assay, compounds #2, #40, #48 and #51 failed to inhibit the bacterially-purified OTUD7B enzyme in catalyzing the cleavage of K11-linked di-ub into mono-ub in vitro (Figure S2A). On the other hand, compound #19 suppressed OTUD7B catalytic activity in this in vitro assay with an IC50 of ~40 μM (under the conditions of 0.5 μM K11-diub and 5 nM active OTUD7B) (Figure 3A,B). In addition, distinct from suppressing OTUD7B activity in vitro (Figure S2B), compound #19 failed to inhibit other tested deubiquitinases including USP21 (Figure 3C,D), and USP9X (Figure S2C), suggesting compound #19 is not a general deubiquitinase inhibitor. Given compound #19 suppresses both OTUD7B-mediated GβL deubiquitination in cells (Figure 2G) and OTUD7B-mediated cleavage of K11-linked di-ub in vitro (Figure 3A), we termed this compound as 7Bi to indicate its potential as an OTUD7B inhibitor. We further confirmed the specificity of 7Bi towards inhibiting OTUD7B by in vitro deubiquitination assays using ubiquitin-AMC as a substrate (without ubiquitin linkage effects), where we observed that 7Bi efficiently inhibited hydrolysis of ubiquitin-AMC in vitro by OTUD7B (Figure 3E), but not its close family member OTUD7A (Figure 3F), nor USP21 (Figure 3G) or USP9X (Figure 3H). To confirm the catalytic inhibitory activity of 7Bi is derived from its hypothesized structure but not contaminants from chemical synthesis, we purified commercially synthesized 7Bi (from 90% purity) by recrystallization to obtain a purity of ~99% (Figure S2D). Compared with commercially synthesized 7Bi with a 90% purity, recrystallization-purified 7Bi retained its ability to suppress OTUD7B-mediated K11-diub cleavage in vitro (Figure 3I); 7Bi-governed the downregulation of Akt-pS473 signals in HEK293 cells (Figure 3J).

### 3.5. 7Bi Treatment Increases GβL Ubiquitination in Cells and Reduces NSCLC Cell Growth

To further examine the effects of 7Bi in cells, we first observed that 7Bi treatment in A549 cells increased K63-linked ubiquitination of GβL at endogenous levels (Figure 3K) presumably through inhibiting OTUD7B. This led to attenuated GβL binding with mTORC2 components including Sin1, Rictor and mTOR (Figure 3L), which is consistent with a critical role of K63-linked GβL ubiquitination in facilitating the mTORC2 kinase complex formation [19]. Consistent with the role of Akt activation in facilitating cell proliferation and survival [20], 7Bi treatment reduced the growth of multiple NSCLC cells including A549 (Figure 3M,N and Figure S2E), H520 (Figure 3O and Figure S2F) and H1299 (Figure 3P and Figure S2G), as well as HEK293 cells (Figure 3Q). Furthermore, 7Bi did not significantly affect OTUD7B protein levels in A549 and HEK293 cells (Figure 2E,F), nor significantly affected OTUD7B binding to GβL (Figure S2H). Together, these data suggest that 7Bi (chemical structure shown in Figure S2I) inhibits OTUD7B deubiquitinase activity in cells and in vitro to suppress cell proliferation. 

Due to the size and side-chain flexibility within the targeted protein-protein interaction site, it is challenging to select a definitive binding pose for 7Bi from the ones suggested by the AtomNet screen. However, some general observations can be made: In the ubiquitin-bound crystal structure that served as a template for our screen, the C-terminal VLRLRG sequence (residues 70–75) of the ubiquitin ligand has several lipophilic as well as charge-charge interactions with the binding site (Figure S2J; light blue, thin sticks). In several of the poses for 7Bi, lipophilic cyclopentyl or methyl groups have similar lipophilic interactions with the surface of the binding site. Likewise, the amide bonds connecting the three central pyrazole rings may mimic the peptide backbone of the ubiquitin. While 7Bi lacks the charged interaction of the endogenous ligand, it can engage in more direct contact with the small sub-pocket near F391. To illustrate the above discussion, an example pose for 7Bi is shown in Figure S2J (yellow, bold sticks).

### 3.6. Genetic and Pharmacological OTUD7B Inactivation Suppresses Leukemia Cell Proliferation

**Figure 4:**
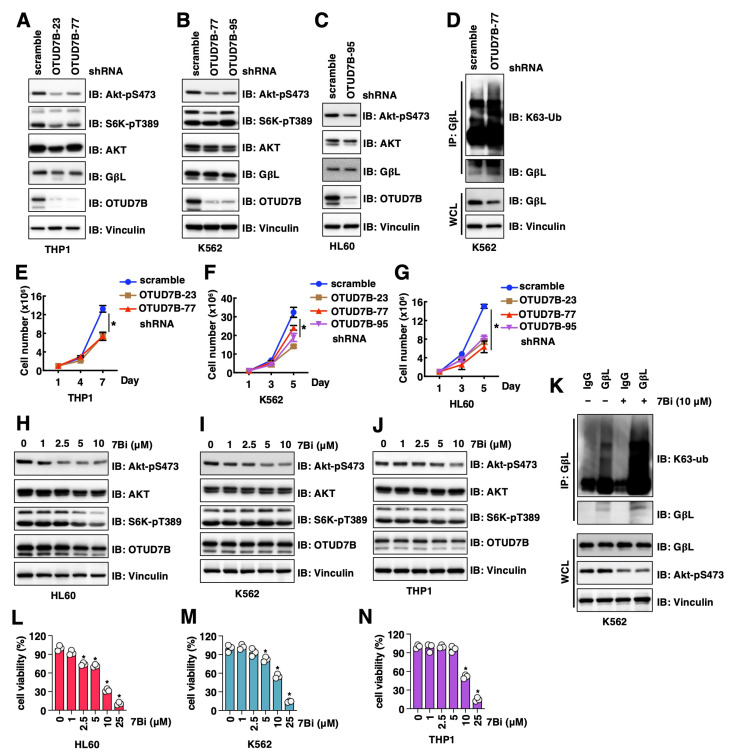
OTUD7B inactivation suppresses leukemia cell proliferation. (**A**–**C**) IB analyses of WCL from indicated cells with depletion of OTUD7B by shRNAs. (**D**) IB analyses of GβL-IP and WCL from indicated K562 cells. (**E**–**G**) Quantifications of cell viability by MTT assays in indicated cells treated with indicated doses of indicated compounds for 72 h. Error bars were calculated as mean ± SD, *n* = 3. * *p* < 0.05 (one-way ANOVA test). (**H**–**J**) IB analyses of WCL from indicated cells treated with indicated doses of 7Bi for 16 h. (**K**) IB analyses of GβL-IP and WCL from K562 cells treated with control or 10 μM 7Bi for 16 h. (**L**–**N**) Quantifications of cell viability by MTT assays in indicated cells treated with indicated doses of indicated compounds for 72 h. Error bars were calculated as mean ± SD, *n* = 3. * *p* < 0.05 (one-way ANOVA test).

In addition to NSCLC, overexpression of OTUD7B was also observed in diffuse large B-cell lymphoma [30] and served as a prognostic marker. This promoted us to examine the roles of OTUD7B in leukemia cells. We firstly depleted OTUD7B in THP1 (monocytes), HL-60 (acute promyelocytic leukemia) and K562 (chronic myelogenous leukemia) cells and found OTUD7B knockdown led to reduced Akt-pS473 signals (Figure 4A–C). Similar to NSCLC cells, depletion of OTUD7B in K562 cells also led to increased K63-linked GβL ubiquitination (Figure 4D). Consistent with a reduction in Akt-pS473 signals, OTUD7B depletion reduced the growth of THP1 (Figure 4E), K562 (Figure 4F) and HL60 (Figure 4G) cells in vitro. More importantly, pharmacological inhibition of OTUD7B by 7Bi reduced Akt-pS473 signals in HL60 (Figure 4H), K562 (Figure 4I) and THP1 (Figure 4J) cells, mimicking the effects of OTUD7B depletion. 7Bi treatment increased K63-linked GβL ubiquitination at endogenous levels in K562 cells (Figure 4K), further supporting OTUD7B may govern leukemia cell proliferation through a similar OTUD7B/GβL/Akt signaling axis as in NSCLC. Consistent with an oncogenic role of OTUD7B in facilitating leukemia cell proliferation, inhibiting OTUD7B by 7Bi reduced the proliferation of HL60 (Figure 4L), K562 (Figure 4M) and THP1 (Figure 4N) cells. Together, these data not only support the critical role of OTUD7B in governing leukemia cell proliferation but also confirm 7Bi as a potential OTUD7B small molecule inhibitor in suppressing leukemia cell proliferation. 

## 4. Discussion

The oncogenic feature of the deubiquitinase OTUD7B has been reported in NSCLC [19]. In this study, we use the AtomNet screening platform to perform an AI-aided virtual compound screening with a chemical library containing 4 million structurally distinctive chemical compounds. The top 59 compounds were purchased and potential OTUD7B inhibitors were further screened by both Akt-pS473 ELISA and Western blotting analyses in NSCLC A549 cells. Overlaying common hits from both screens narrowed down candidates to 10 hits, and among them, two compounds (#19 and #51) survived after another two tiers of selection, including (1) exerting an ability to reduce Akt-pS473 in at least three out of four NSCLC cell lines and (2) suppressing NSCLC cell growth in vitro. Both compounds were able to block OTUD7B-mediated GβL deubiquitination in cells. However, in in vitro OTUD7B deubiquitnation assays using K11-linked diUb as a substrate, only one compound #19- we termed as 7Bi- showed an inhibitory effect, suggesting the other compound likely indirectly regulated GβL-ubiquitination and Akt-pS473 signals in cells. We further confirmed that 7Bi treatment efficiently reduced the growth of multiple NSCLC cells and HEK293 cells. We further found OTUD7B also governed various leukemia cell proliferation and 7Bi treatment efficiently reduced leukemia cell proliferation in vitro. Together, our study identifies a small molecule 7Bi as a possible OTUD7B catalytic inhibitor, which may be a viable therapeutic agent in treating cancers with OTUD7B overexpression including breast cancer [28], lung cancer [29], diffuse large B-cell lymphoma [30] and NSCLC [31], as well as other human disorders including myocardial infarction [32]. To the best of our knowledge, this is the first non-covalent small molecule inhibitor of OTUD7B.

Notably, the IC50 values for 7Bi to inhibit Akt-pS473 signals in NSCLC cells range from 1 to 10 μM and the IC50 values for 7Bi to suppress NSCLC cell growth are ~2.5 μM, thus further in-depth SAR (structure-activity relationship) studies are necessary to improve its potency. In addition, the IC50 value for #19 in this in vitro OTUD7B deubquitination assay is determined by the amounts of di-ub (substrate) and OTUD7B, as well as the reaction time, thus it does not provide a comparable evaluation to its in-cell IC50 values. In addition, although 7Bi does not inhibit OTUD7A, USP21 and USP9X in vitro, the specificity of 7Bi over a broader range of DUBs remains to be further determined. Our current in-cell and in vitro evaluations suggest 7Bi as a potential OTUD7B inhibitor while the target engagement has not been tested and further in-depth analyses are required. Moreover, the effects of 7Bi on tumor growth in animal models, as well as the bioavailability and pharmacological properties of 7Bi also need to be profiled and examined. Nonetheless, the identification of 7Bi as the first small molecule inhibitor of OTUD7B is a start for the development of viable therapeutic agents targeting OTUD7B. We have searched Scifinder for literature or patents on 7Bi, and there were none. We also found no reported bioactivities for closely related compounds. In addition, AtomNet seems to be able to find distinct sets of compound inhibitors for closely related deubiquitinase members such as OTUD7A [35] and OTUD7B (this study). Cross-activity examinations confirmed that 7Ai does not significantly suppress OTUD7B activity in cells [35] and 7Bi does not inhibit OTUD7A activity in vitro (Figure 3F). However, due to the nature of AtomNet, it is difficult to generate high quality simulation structures to reveal reliable binding pose of 7Bi that can guide mutagenesis studies for further validation. This will require solving the structure of 7Bi bound to OTUD7B.

Given that OTUD7B exerts both deubiquitinase dependent- and independent-functions in tumorigenesis and immune regulation, the identification of OTUD7B catalytic inhibitors or protein-protein interaction inhibitors may lead to the further development of OTUD7B-PROTACs or DUBTACs by using discovered OTUD7B binding chemical structures, which could further enhance OTUD7B targeted therapeutic efficacy and broaden its potential applications in disease treatment. Notably, considering the immune regulatory function of OTUD7B that OTUD7B may facilitate T cell activation [26], the effects of OTUD7B inhibitors in possibly creating an immune suppressive micro-environment warrants further in-depth investigations and if so cautions should be taken when OTUD7B inhibition is used in the clinic.

## 5. Conclusions

In this study aided by Atomnet, we screened 4 million compounds and identified a potential OTUD7B catalytic inhibitor that we term as 7Bi. 7Bi treatment in NSCLC and HEK293 cells efficiently reduced Akt-pS473 signals by interfering with OTUD7B-directed GβL deubiquitination, leading to reduced cell growth. In addition, we report an oncogenic function for OTUD7B in leukemia by similarly controlling K63-linked GβL ubiquitination and mTORC2 activity in governing leukemia cell proliferation. We further demonstrate that 7Bi also efficiently suppresses leukemia cell proliferation. Together, our study provides the chemical structure for a possible OTUD7B inhibitor that can be further improved by medicinal chemistry or PROTAC/DUBTAC approaches, with potential as viable therapeutic agents in treating cancer with OTUD7B overexpression. Additional studies to determine the binding affinity and structural insights would be helpful for the next rounds of SAR studies that aim to improve the potency and specificity of 7Bi. Further, PK/PD studies to examine and improve the chemical properties of 7Bi, and more importantly, examination of the effects of 7Bi or improved 7Bi in suppressing OTUD7B activity and oncogenic function in murine cancer models would be needed to gain insights for its therapeutic potential in pre-clinical models.

## 6. Patents

A provision patent application is being submitted.

## Data Availability

The identity and applications of 7Bi have been submitted for a provision patent application.

## Supplementary Materials

The following supporting information can be downloaded at: www.mdpi.com/article/10.3390/cancers15020517/s1, Figure S1: Testing effects of synthesized compounds on inhibiting Akt-pS473 signals in cells; Figure S2: Validation of possible OTUD7B inhibitors in in vitro OTUD7B catalytic assays.
