# Supplementary material for: AtomNet-Aided OTUD7B Inhibitor Discovery and Validation
Source: Cancers (Basel). 2023 Jan 14;15(2):517. doi: 10.3390/cancers15020517 (PMC9856706; doi:10.3390/cancers15020517)
Supplement: Supplementary file 1 [file cancers-15-00517-s001.zip › cancers-2079927-supplementary.pdf]

Supplementary Materials of AtomNet-aided OTUD7B inhibitor discovery and validation

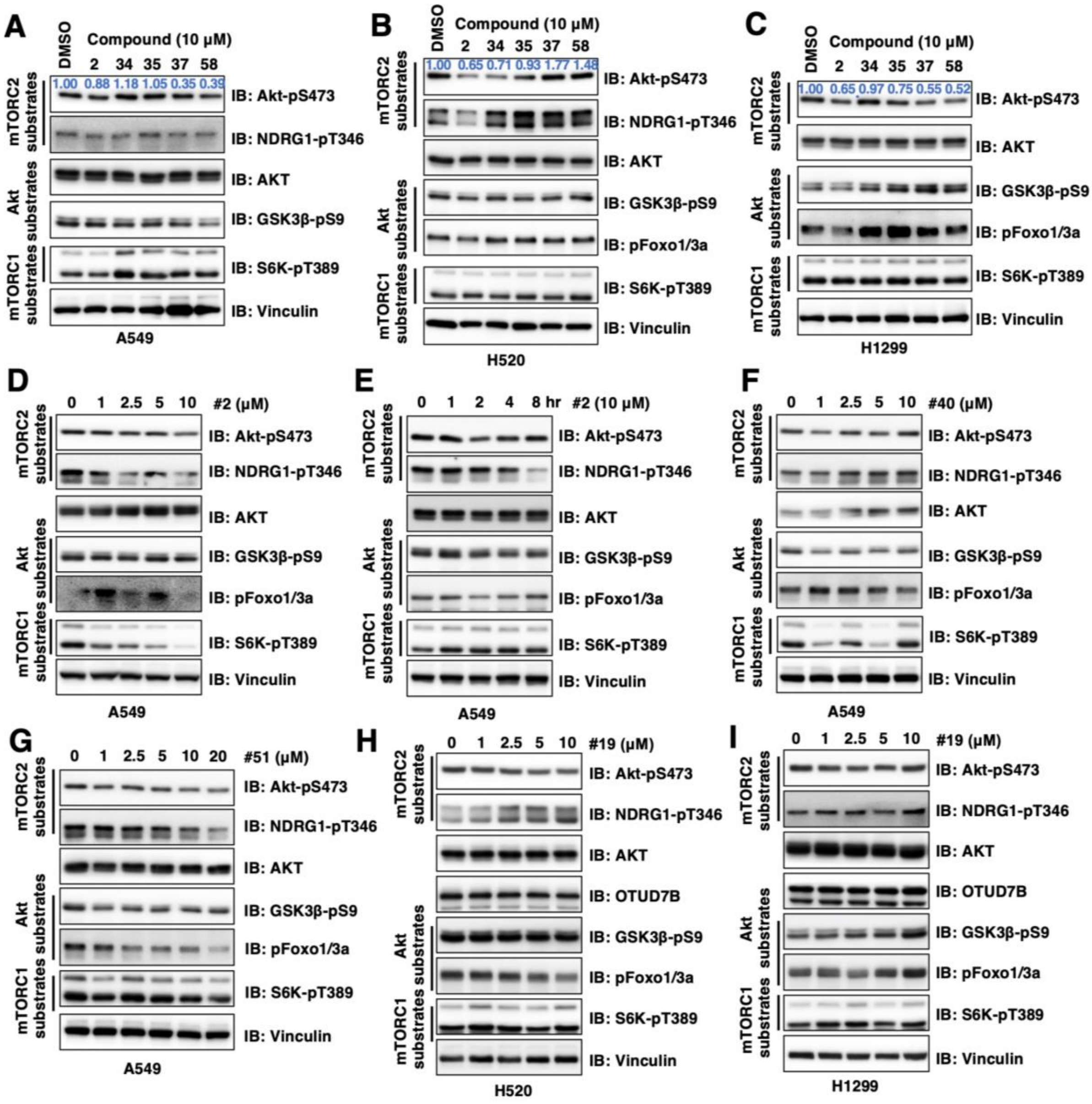

Supplemental Figure S1. Testing effects of synthesized compounds on inhibiting Akt-pS473 signals in cells. (A–C) IB analyses of WCL from indicated cells treated with 10 μM of indicated compounds for 16 hrs. (D–I) IB analyses of WCL from indicated cells treated with indicated doses of compounds for 16 hrs.

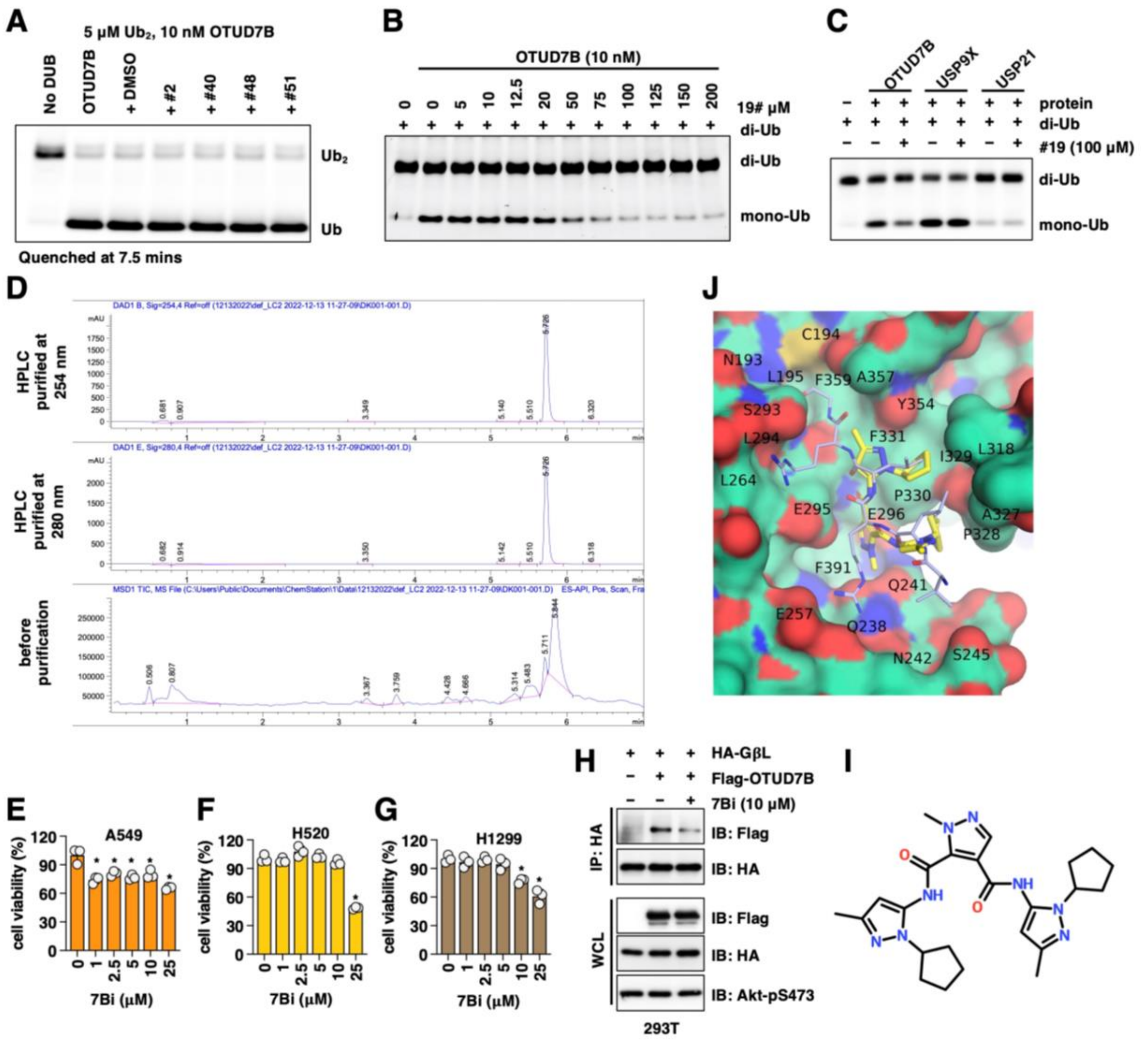

Supplemental Figure S2. Validation of possible OTUD7B inhibitors in in vitro OTUD7B catalytic assays. (A,B) In vitro OTUD7B deubiquitination assays using K11-diub incubated with recombinant active OTUD7B in the presence of indicated doses of compounds. (C) In vitro DUB assays using indicated DUB incubated with K11-diub. (D) Determination of the purity of 7Bi from Mcule (90%), bottom and recrystallized 7Bi (99%) at either 280 nm or 254 nm wavelength as indicated. (E–G) Quantifications of cell viability by MTT assays in indicated cells treated with indicated doses of indicated compounds for 72 hrs. Error bars were calculated as mean+/-SD, n=3. * $p < 0.05$ (one-way ANOVA test). (H) IB analysis of HA-IP and WCL from HEK293T cells transfected with indicated DNA constructs. (I) The chemical structure of 7Bi. (J) A potential binding pose for 7Bi (yellow, bold lines) at the distal S1 ubiquitin-binding site (PDB ID 5LRW, green). Nearby protein residues, including the catalytic C194, are labeled. The C-terminal VLRLRG substructure (residues 70-75) sequence of the co-crystallized ubiquitin is shown for reference (light blue, thin sticks).
